# “Galectin-1 Induces Central and Peripheral Cell Death: Implications in T-Cell Physiopathology”

**DOI:** 10.1155/2000/36321

**Published:** 2000

**Authors:** C. E. Sotomayor, G. A. Rabinovich

**Affiliations:** 1 Inmunología. Dpto. Bioquímica Clínica Facultad de Ciencias Químicas Universidad Nacional de Córdoba Córdoba Argentina; 2 Laboratorio de Inmunología Departamento de Bioquímica Clínica Facultad de Ciencias Químicas Universidad Nacional de Córdoba, Ciudad Universitaria Córdoba 5000 Argentina

**Keywords:** galectin-1, apoptosis, immunomodulation, macrophages, autoimmunity

## Abstract

The immune system has a remarkable capacity to maintain a state of equilibrium even as it responds to a diverse array of foreign proteins and despite its contact exposure to self-antigens. Apoptosis is one of the mechanisms aimed at preserving the homeostasis after the completion of an immune response, thus returning the immune system to a basal state and warranting the elimination of autoagressive cells in both central and peripheral lymphoid organs. Targeted deletions in critical genes involved in the apoptotic death machinery together with natural spontaneous mutations have clearly shown the importance of apoptosis in the regulation of the immune response. This complex scenario of stimulatory and inhibitory genes has been enriched with the finding that galectin-1, a 14.5 kDa β-galactoside-binding protein, is able to induce apoptosis of immature cortical thymocytes and mature T cells by cross-linking cell surface glycoconjugates. Galectin-1 is present not only in central and peripheral lymphoid organs, but also at sites of immune privilege. In the present article we will discuss the implications of galectin-1-induced apoptosis in T-cell physiopathology in an attempt to validate its therapeutic potential in autoimmune and inflammatory diseases.


*Developmental Immunology*, 2000, Vol. 7(2-4), pp. 117-129
Reprints available directly from the publisher
Photocopying permitted by license only

© 2000 OPA (Overseas Publishers Association)
N.V. Published by license under the
Harwood Academic Publishers imprint,
part of the Gordon and Breach Publishing Group.
Printed in Malaysia

### "Galectin-1 Induces Central and Peripheral Cell Death: Implications in T-Cell Physiopathology"

C.E. SOTOMAYOR* and G.A. RABINOVICH†

*Inmunología. Dpto. Bioquímica Clínica Facultad de Ciencias Químicas. Universidad Nacional de Córdoba. Córdoba. Argentina*

The immune system has a remarkable capacity to maintain a state of equilibrium even as it responds to a diverse array of foreign proteins and despite its contact exposure to self-antigens. Apoptosis is one of the mechanisms aimed at preserving the homeostasis after the completion of an immune response, thus returning the immune system to a basal state and warranting the elimination of autoagressive cells in both central and peripheral lymphoid organs. Targeted deletions in critical genes involved in the apoptotic death machinery together with natural spontaneous mutations have clearly shown the importance of apoptosis in the regulation of the immune response. This complex scenario of stimulatory and inhibitory genes has been enriched with the finding that galectin-1, a 14.5 kDa β-galactoside-binding protein, is able to induce apoptosis of immature cortical thymocytes and mature T cells by cross-linking cell surface glycoconjugates. Galectin-1 is present not only in central and peripheral lymphoid organs, but also at sites of immune privilege. In the present article we will discuss the implications of galectin-1-induced apoptosis in T-cell physiopathology in an attempt to validate its therapeutic potential in autoimmune and inflammatory diseases.

*Keywords:* galectin-1, apoptosis, immunomodulation, macrophages, autoimmunity

### INTRODUCTION

Death, along with growth and differentiation, is a critical part of the life cycle of the cell. Homeostasis control of cell number is thought to be the result of the dynamic balance between cell proliferation and cell death. It is only in the past ten years, that the attention has been focused on the physiological occurrence of cell death and its role in the homeostasis.

Apoptosis or programmed cell death, is a phenomenon that plays a crucial role in a myriad of physiological and pathological processes. This review will briefly cover some relevant aspects of programmed cell death in the immune system in an attempt to provide valuable information about new molecules responsible for triggering death signals, such as galectin-1. The implications of this protein will be discussed in the context of T cell physiology and the regulation of central and peripheral tolerance. Finally, novel and intriguing findings will also be discussed implicating the use of this carbohydrate-binding protein in the treatment of autoimmune and inflammatory diseases.

* Addressed correspondence: Dra. Claudia E Sotomayor, Laboratorio de Inmunología. Departamento de Bioquímica Clínica. Facultad de Ciencias Químicas. Universidad Nacional de Córdoba. Ciudad Universitaria. (5000) Córdoba. Argentina. Phone # 54–0351–4334164. Fax # 54–0351–4334187. E-mail: csotomay@bioclin.fcq.unc.edu.ar
† Both authors contributed equally to this article.

### APOPTOSIS: A PHYSIOLOGICAL MECHANISM OF HOMEOSTASIS AND SELF-TOLERANCE IN THE IMMUNE SYSTEM

Physiological cell death by activation of intrinsic cell suicide program, provides an efficient and critical control point for eliminating unwanted cells. This type of regulation allows the elimination of cells that have been produced in excess, have developed improperly, or have sustained genetic damage (Schwartzman and Cidlowski, 1993, Thompson, 1995). As to the initiation of the death program, the decision of a cell to undergo apoptosis can be influenced by a wide variety of extrinsic and intrinsic regulatory stimuli (Thompson, 1995). On the other hand, the effector stage takes place after triggering a number of evolutionarilly conserved genes that regulate a final common cell death pathway, that is preserved from invertebrates to humans (Vaux el al., 1994; Raff, 1992).

Programmed cell death is an important physiological process acting both during development and homeostasis. Aberrations in this process are implicated in a broad range of diseases. While loss of apoptotic response can lead to cancer or autoimmune diseases, an increased apoptotic rate is implicated in neurodegenerative diseases, brain ischaemia and myocardial infarction. In this context, the immune system offers an excellent scenario for the discussion of the relevance of apoptosis in the development and maintenance of homeostasis. Immunologists have focussed largely on defining the stimuli that induce growth, differentiation and effector functions of lymphocytes and over the last two decades, the essential feature of lymphocyte activation and immune responses have been defined in considerable detail. Less is known, however, about the mechanisms that terminate immune responses. Mechanisms such as apoptosis are important after a productive immune response to a foreign antigen, when the immune system is returned to a state of rest (van Parijs and Abbas, 1998). This process allows the immune system to respond effectively to a new antigenic challenge. Moreover, apoptosis is crucial for achieving self-tolerance avoiding the development of receptors capable of recognizing self-antigens. Elucidating the nature of these homeostatic mechanisms may lead to better strategies for suppressing harmful immune responses and for augmenting and sustaining beneficial responses to microbial vaccines and tumors.

### Death Signals in T-lymphocytes Development

The development of T cells is governed by the microenvironment of the thymus. A large number of precursor cells migrate into the thymus daily, where they are subjected to selection in a critical process of thymic education. Distinct stages of the T cell differentiation and maturation within the thymus have been identified and associated with the cells surface expression of molecules such as CD4, CD8 and the TCR/CD3 complex. In the subset of CD4+ CD8+ double-positive (DP) lymphocytes, more than 95% of cells are destined to die by positive and negative selection. The majority of DP thymocytes fail to generate a functional TCR that successfully interacts with major histocompatibility complex proteins (MCH) and consequently die by a process called death by neglect. Those cells bearing TCR that recognize MHC with intermediated affinity are positively selected. Moreover, a subset of DP cells recognizing MHC molecules with high affinity are subjected to negative selection and consequently deleted by apoptosis (Surh and Sprent, 1994). A high percentage of the thymocytes generated daily die within the thymus. Hence, massive cell death is a crucial part of T-cell development, as reflected by the fact that each new T cell undergoes for self-MHC restriction and self-tolerance. Thymocytes that success fully pass through positive and negative selections down-regulated the expression of either CD4 or CD8 and differentiate into functional single-positive cells and go out as mature T cells to the periphery.

Apoptosis in DP thymocytes is also triggered by glucocorticoids (Surh and Sprent, 1994). The induction of cell death in thymocytes by glucocorticoids was one of the first systems studied by Wyllie (1980) and Cohen et al. (1992). This work provides an early model of cell death by a specific signaling molecule and was fundamental to the development of the concept of active cell death as a cellular response rather than a passive act of overwhelming cell damage. Thymocytes, in addition to T-cells lines, are highly sensitive to apoptosis induced by exposure to endogenous glucocorticoids (Sprent et al., 1988) in an active process, requiring *de novo* gene expression (Wyllie, 1980). In this sense, Ashwell and colleagues performed *in vivo* experiments ( Vacchio et al., 1994, King et al., 1995) demonstrating that a subset of thymic epithelial cells are steroidogenic and that inhibition of steroid synthesis modified the profile of lymphoid thymic populations. Furthermore, data obtained by the creation of transgenic lines of mice carrying a glucocorticoid receptor antisense (King et al., 1995), indicated that thymocytes that do not bind to MHC are deleted by endogenous steroids.

Elimination of autorreactive lymphocytes may occur via activation-induced cell death: the same signals that trigger activation of peripheral mature T cells induce apoptosis of thymocytes (Takayama and Sitkovsky, 1989). The difference between positive selection of normally functional immature T cells and elimination of autorreactive cells may lie in the affinity of their TCRs for self antigens and increasing evidence suggest that co-stimulatory signals could play an important role in this process ( Takayama and Sitkovsky, 1989, Iwatw et al., 1992, Migliorati et al., 1992).

In the network of intrathymic signals, the positive selection might also result from antagonism between glucocorticoid and activation-induced death. This model clearly demonstrates that thymocytes unable to bind to the MHC are eliminated via glucocorticoid signals, those cells that engage T cell receptor complex with moderate avidity are protected via the steroid/ TCR antagonism, whereas thymocytes able to transduce TCR signals of sufficient strength, are eliminated via activation-induced cell death.

### The Fas Death Factor: "Turning Mature Lymphocytes Off"

Among the most important molecules involved in triggering apoptosis appears the cell surface receptor Fas, also called APO-1 or CD95, which induced apoptosis upon binding to its natural ligand FasL, APO-1L, or CD95L, or specific agonist antibodies (Nagata, 1997). This death receptor is a member of the tumor necrosis factor (TNF) and nerve growth factor (NGF) receptor superfamily, which among others, also includes TNF-R1, TNF-R2, low-affinity NGFR, CD40 and CD30. While family members are defined by the presence of cysteine-rich repeats in their extracellular domains, CD95 and TNF-R1 also share an intracellular region of homology, designated the "death domain", which is required to signal apoptosis.

Fas, a 36 kDa type I membrane protein, is expressed on a wide variety of cell types including hematopoietic and epithelial cells. Expression of Fas on T and B-lymphocytes increases after antigen receptor-mediated activation (Nishimura et al., 1995). This molecule is also expressed in cells transformed with human T-cell leukaemia virus (HTLV-1), human immunodeficiency virus (HIV) or Epstein-Barr virus (EBV). Many nonlymphoid tissues, such as liver, also express Fas (Nagata, 1997; Galle et al., 1995).

Fas-L is a 40 kDa type II transmembrane protein that mediates apoptosis by crosslinking of Fas in sensitive target cells and in contrast to the widespread distribution of Fas, its ligand exhibits a highly restricted pattern of expression. FasL is induced on mature CD4+ and CD8+ T lymphocytes following activation, but it is not expressed by other hematopoietic cells (Tanaka et al., 1995; Suda et al., 1995). This molecule has been shown to play a role in the maintenance of peripheral T- and B-cell homeostasis. Noteworthy, in some circumstances FasL can be proteolitically cleaved from the membrane by a metalloproteinase, occuring in a soluble form that can act as a cytotoxic effector molecule or an inhibitor of apoptosis (Strand and Galle, 1998).

The Fas-mediated apoptotic cascade is initiated by the direct association of the death receptor Fas with the adapter molecule FADD (Fas-associated protein with death domain) and the effector protease FLICE (FADD homologous ICE/ CED-3-like prtease, FADD-like ICE), a member of the ICE/ Ced-3/ caspase family (Henkart, 1996). The assembly of this signalling complex triggers the caspase cascade. The

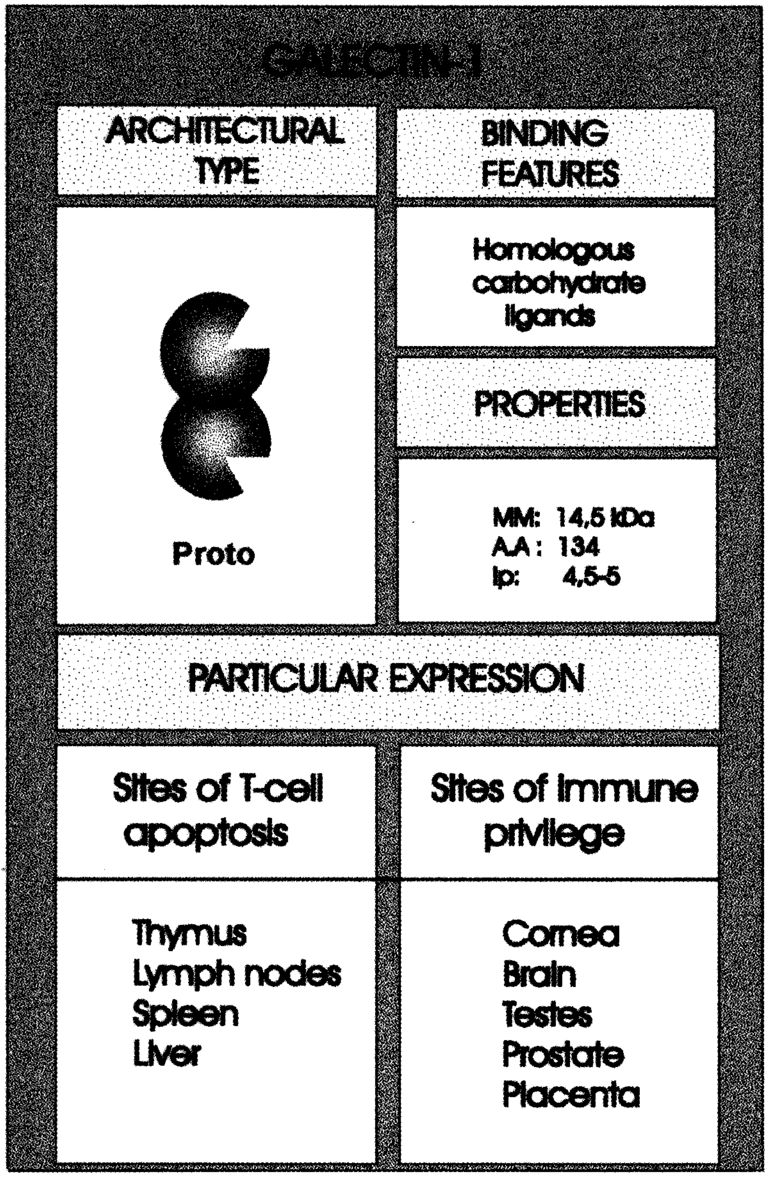

MM:Molecular mass, A.A: Amino acid residues, Ip: Isoelectric point

FIGURE 1 Galectin-1, characteristic and particular expression activated caspase members can cleave various substrates resulting in characteristic apoptotic morphology of cytoplasm and nuclei.

Activated T-cells express Fas as well as FasL. Although they use FasL to kill their targets, they can also use this molecular weapon against each other to limit their own number protecting activated mature T cells from continued secretion of potentially harmful levels of cytokines. These cells are eliminated from the circulation by activation of the cell death program (Russel et al., 1993; Singer and Abbas, 1994).

### Role of Fas-mediated cell death in the suppression of immune response, self-tolerance and autoimmunity

Activation-induced apoptosis of mature T cells occurs via Fas and Fas ligand (FasL) interactions. A set of in vitro experiences using cell lines or T-cells hybrids provided successful information about the relevance of Fas death signals (Bruner et al., 1995; Dhein et al., 1995; Ju et al., 1995). In the recent years, much work has focused on the molecular mechanisms by which these molecules regulate apoptosis specially the relationship of Fas-FasL interaction with members of the Bcl-2 family in the context of peripheral immune tolerance (van Parijs et al., 1998).

van Parijs and colleagues clearly demonstrated that Bcl-2 protects T cells from apoptosis caused by the absence of growth factors and activation stimuli, a process called passive cell death and prolongs the response of T cells to a model of foreign antigen *in vivo*. In contrast, Fas induces apoptosis in autorreactive T cells or in activated T cells that are restimulated with high concentrations of antigen by a process called activation-induced cell death. *In vivo*, Fas-mediated apoptosis is responsible for eliminating T cells responding to a model systemic self-antigen and for preventing autorreactive helper T cells from activating self-reactive B cells.

Genetic defects that predispose to autoimmunity are providing valuable information about the mechanisms responsible for terminating T-cell responses to self-antigens. In this sense, the importance of Fas/FasL interaction in peripheral tolerance, has been highlighted by the MRL-*lpr/lpr* or C3H-*gld/gld* mice strains which carry spontaneous mutations in Fas and FasL genes respectively. These mice exhibit multiple autoimmune systemic disorders characterized by the presence of autoantibodies, hypergammaglobulinemia and immune complex nephritis, all features of a lupus-like syndrome (Russell et al., 1993; Russell and Wang, 1993). Nagata and colleagues (Adachi et al., 1995) created a Fas-/- mice by targeted deletion of the Fas gene. These mice displayed enhanced and accelerated lymphoproliferation in comparison to *lpr/lpr* mice (Adachi et al., 1995).

Recently, a human syndrome of autoimmunity associated lymphadenophathy has been described, carrying various inherited abnormalities in Fas-mediated killing. These abnormalities include the inheritance of two mutant Fas alleles and unknown signalling defects (Fisher et al., 1995; Rieux-Laucat et al., 1995). It is likely that other alteration in Fas or downstream signalling intermediates will be identified as cause of autoimmune syndromes. Recently, an inhereted human caspase 10 mutation has been described showing defective lymphocyte and dendritic cell apoptosis in autoimmune lymphoproliferative syndrome (type II) (Wang et al., 1999).

The elucidation of the mechanisms involved in T cell survival *in vivo* will lead to rational approaches for controlling autorreactivity, while enhancing immunological memory.

### Role of Fas-mediated cell death in immunological privileges tissues

The immune privileged tissues are vulnerable sites in the body where even minor cellular immune reactions and their associated inflammatory response can cause irreversible damage. Therefore, protective mechanisms are required to avoid unwanted immune reactions that could result in impaired organ functions. Interestingly, not only are these "immune privileged sites" protected against overwhelming inflammatory responses, but they can also support allogenic or xenogenic tissue grafts. Some explanation about how immune privileged is maintained, involve physical barriers and cytokines profiles (Streilin, 1993).

FasL has been also reported to be constitutively expressed in two immunologically privileged tissues, such as the eye and the testis. Griffith et al. (1995) showed that the constitutive expression of FasL on parenchymal cells within the anterior chamber of the eyes can maintain the integrity of this immune-privileged site. It can be reported that Fas+ lymphoma cells can be induced to undergo apoptosis when exposed *in vitro* to explants of cornea and iris-ciliary body from eyes of normal mice, but not when exposed to eyes of *gld* mice, which do not express FasL. Interestingly, FasL has been found to be expressed in Sertoli cells of the testis (Bellgrau, 1995).

Taken together, these finding unequivocally suggest that FasL plays a crucial role by avoiding the damage inflicted by activated T cells to these tissues. The expression of high levels of FasL represents a defensive mechanism to prevent damage caused by inflammation through an induction of apoptosis of activated cells expressing elevated levels of Fas antigen (Osborne, 1996).

Recent data confirm that expression of functional FasL might confer the status of immune privilege to tumor cells, representing an active defense mechanism resulting in the elimination of immune competent anti-tumor lymphocytes (O'Connell et al., 1999). In this sense, FasL expression could be associated with the later tumor recurrence which is commonly observed in several tumors such as melanomas. These tumors often recur after 20 years or more, and this can be explained by a loss of immunosurveillance. Hence, FasL expression might contribute to tumor progression, invasion or metastasis.

### GALECTINS: A FAMILY OF CARBOHYDRATE-BINDING PROTEINS WITH IMMUNOREGULATORY PROPERTIES

### Definition and Background

Galectins are a growing family of animal β-galactoside binding proteins, defined by two common characteristics: (a) affinity for poly-*N*-acetyllactosamine-enriched glycoconjugates and (b) significant sequence homology in the carbohydrate binding site (Barondes et al., 1994a; Barondes et al., 1994b). In the past few years, there has been progress in identifying new galectins in mammals and other species, cloning them and ascertaining the structural features that determine carbohydrate binding. Ten mammalian galectins have been well characterized and studied (Leffler, 1997). Structural analyses of various galectins indicate the presence of homodimers of carbohydrate-binding domains in galectin-1 and galectin-2, a monomer of the carbohydrate-binding domain in galectin-5 and a single polypeptide chain with two carbohydrate-binding domains joined by a link peptide in galectins-4, –6, –8 and –9. Galectin-3 has a carbohydrate-binding domain, a short N-terminal segment, consisting of PGAYPG (X) repeats and an intervening stretch of amino acids, enriched with proline, glycine and tyrosine. Expression analysis have revealed that certain galectins display a restricted distribution, e.g. galectin-2 in hepatoma, galectin-4 in small intestine, galectin-5 in erythrocytes and galectin-7 in keratinocytes. Galectins with a broad tissue distribution include galectin-1, expressed in cardiac, smooth and skeletal muscle, macrophages, neurons, thymus, kidney and placenta, galectin-3 present in blood cells such as monocytes, mast cells, and tumor cells and galectin-8 expressed in liver, kidney, cardiac muscle, lung and brain (Rabinovich, 1999). Extensively studied among them is galectin-1, an homodimer with an Mr of approximately 14,500 Da. It has been postulated that this protein recognizes a wide variety of extracellular receptors such as fibronectin (Ozeki et al., 1995) and laminin (Zhou and Cummings, 1993) and cell surface glycoproteins such as CD45 and CD43 (Baum et al., 1995a, Perillo et al., 1995) through deciphering specific glycocodes (Kasai and Hirabayashi, 1996).

By virtue of this specific recognition, this evolutionarily conserved family of animal lectins have been implicated in a variety of functions that include cell growth regulation (Sandford and Harris-Hooker, 1990; Wells and Mallucci, 1991), cell adhesion (Cooper et al., 1991; Zhou and Cummings, 1993; Rabinovich et al., 1999a), neoplastic transformation (Akahani et al., 1997a), immune responses (Ofner et al., 1990; Levy et al., 1983) and T-cell apoptosis (Perillo et al., 1995; Rabinovich et al., 1998; Iglesias et al., 1998a). However, the widespread expression of multiple members of the galectin family and presumed overlaps in carbohydrate-binding specificities have made it difficult to establish the *in vivo* function of individual members of this class of proteins (Poirier and Robertson, 1993).

All known members of this family lack a signal peptide (Barondes et al.,1994a), are found in the cytosol and are isolated as soluble proteins. However, there is evidence that some members are externalized by an atypical secretory mechanism (Cooper and Barondes, 1990).

The expression pattern of different galectins changes during development (Colnot et al., 1997) and this pattern is also altered at sites of inflammation and in breast, colon, prostate and thyroid carcinomas (Akahani, et al. 1997b). The level of expression of some galectins by tumor cells has been show to be correlated with metastatic potential. Although galectins exert their effects through recognition of a spectrum of appropriately glycosylated proteins on the surface of a variety of cells, the precise mechanism and signal transduction pathways involved in these functions remain largely unknown.

### GALECTIN-1: EXPRESSION WITHIN THE IMMUNE SYSTEM AND IMPLICATIONS IN T-CELL PHYSIOLOGY

### Participation of Galectin-1 as a Gear of the Central and Peripheral Cell Death Machinery

Galectin-1 has been shown to be expressed in sites where T-cell apoptosis takes places including the thymus (Baum et al., 1995a), spleen (Rabinovich et al., 1996) and lymph nodes (Baum, et al., 1995b). It has been particularly found in thymic epithelial cells (Baum et al., 1995a), activated macrophages (Rabinovich, et al., 1998) and effector T cells (Blaser, et al., 1998) (Figure 1).

The first evidence suggesting that galectin-1 could be involved in central immune tolerance was first suggested by Goldstone and Lavin (1991), who reported an increase in the levels of galectin-1 mRNA during apoptosis induced by glucocorticoids. As clearly stated, the interplay between thymic steroids and TCR signals modulate cell death within the thymus (Wyllie, 1980). It is well known that thymocyte maturation also requires the participation thymic epithelial cells and extracellular matrix components (Anderson et al., 1994; Anderson et al., 1996). In this sense, Baum et al. (1995a) demonstrated that human thymic epithelial (TE) cells produced high levels of galectin-1 which bound specifically to the surface of cortical thymocytes. This endogenous lectin mediated the adhesion of thymocytes to TE cells. Sensitivity of T cells to galectin-1 was found to be modulated by the expression of glycosiltransferase enzymes that might modify the availability of oligosaccharide ligands for galectin-1. Perillo et al (1997) provided then concluding evidence that galectin-1 induced apoptosis of two distinct subpopulations of non-selected and negatively-selected CD4low, CD8low immature cortical thymocytes (Perillo et al., 1997). Null mutant mice in galectin-1 gene will be useful to confirm whether galectin-1 plays a critical role in the central cell death machinery for postive and negative selection of developing thymocytes.

Activation-induced cell death of mature T cells is one of the mechanisms aimed at turning off the immune response and preventing the expansion of autoagresive clones. In addition to its role in central tolerance, Perillo et al. (1995) clearly showed that galectin-1 induced apoptosis also in activated mature T cells. Recently, Blaser et al. (1998) found that galectin-1 expression was strongly up-regulated in effector T cells and inhibited antigen-induced proliferation of naive and memory CD8+ T cells. This mechanism was mediated by an arrest in cell cycle progression at the level of S and G2/M stages (Allione et al., 1998).

Moreover, we have recently shown the presence of a galectin-1-like protein, which was differentially regulated in resident, inflammatory and activated macrophages (Rabinovich et al., 1996). Total and surface expression of this carbohydrate-binding protein, called RMGal (for rat macrophage galectin-1) were found to be up-regulated when these cells were activated with protein kinase C activators such as phorbol esters (PMA) and chemotactic peptides (fMLP). When this protein was purified by affinity chromatography and its biochemical properties and amino acid sequence were determined, a definitive conclusion was reached concerning its pertenence to the galectin-1 subfamily ( Rabinovich et al., 1998).

Macrophages play an important role in several steps of innate and adaptive immune response. While they have great phagocytic ability and a large repertoire of lytic enzymes and secretory products, they also express a wide array of cytokines, surface receptors able to recognize specific antigen epitopes. Activated macrophages are more efficient in their ability to process and present antigens in the initiation of an immune response by virtue of the higher levels of major histocompatibility complex molecules (Adams and Hamilton, 1984; Adams et al. 1996). Moreover, they are also key immunoregulatory cells able to turn off an established immune response (Aliprantis et al., 1996). We determined that by using current techniques to evaluate apoptosis, such as DNA fragmentation, TUNEL assay and transmission electron microscopy that galectin-1 produced by activated macrophages is able to induce apoptosis of mature T cells in a carbohydrate-dependent manner (Rabinovich et al., 1998). The results were comparatively stronger to those found in an heterologous system using CLL-I, the 16 kDa chicken isolectin (Rabinovich et al., 1997).

RMGal protein was found to be secreted only when macrophages were activated with potent biochemical agents and pro-inflammatory cytokines (Rabinovich et al., 1999c).

### Galectin-1 Expression in Immune Privileged Sites: a Novel Mechanism of Protection?

Galectin-1 is also present in sites of immune privilege, such as placenta (Hirabayashi and Kasai, 1988; Iglesias et al., 1998a), cornea (Ogden et al., 1998) and prostate (Allen et al., 1991; Hirabayashi and Kasai, 1993). The presence of this protein in these vulnerable sites might contribute to mantain a state of tolerance by inducing apoptosis of inflammatory and activated T cells that could provoke injury, autoimmune damage or infection. Accordingly, galectin-1 could also be proposed as an alternative regulatory signal to regulate immune privilege. Expression of this protein in first term gestation placenta would prevent inflammatory T cells from harming the fetus (Iglesias et al., 1998a). In agreement, a protein related to the galectin family called GRIFIN (galected-related interfiber protein) has been recently identified in lens, cellular structures of the optical system (Ogden et al. 1998). Furthermore, recent results reported by Maldonado et al. (1999) have clearly shown by using immunogold techniques, that galectin-1 is expressed in Müller cells in post-natal chicken retina and in mitochondria localized in the inner segments of cone cells. Expression of this protein in these glial cells suggests a potential role in metabolic and immunomodulatory processes between Müller and other retinal cells.

This pro-apoptotic protein was found to be up-regulated by metastatic in comparison to non-invasive tumors. In certain way, tumors might be considered as immune privileged sites and several mechanisms for tumor evasion of immune recognition have been proposed, such as decreased expression of MHC class I or B7.1 co-stimulatory signal, TGF-β secretion, endocytosis of tumor antigens and FasL expression (O'Connell et al., 1999). In this sense, one should suspect that galectins in tumor cells can trigger apoptosis of tumor-infiltrating lymphocytes (TILs), thus allowing the tumor to escape immune attack.

### Death Signals in the Periphery

Despite striking similarities in their localization, critical differences should be distinguished between FasL and galectin-1. FasL induces apoptosis by a interaction with its counterpart Fas/APO-1/CD95 within the same cell (suicide) or a neighbour cell (fraticide), while galectin-1 is secreted and binds to cell surface glycoconjugates (Perillo et al., 1997) on cortical thymocytes and T cells. Besides, galectin-1 and FasL apparently use different signal transduction pathways to engage the apoptotic program of the cell. Recently, Su et al. (1996) and Perillo et al. (1995) showed that the T lymphoblastoid cell line MOLT-4 that was unsensitive to FasL-induced apoptosis, was susceptible to galectin-1. In contrast, the T lymphoblastoid cell line CEM, which was sensitive to FasL was resistant to galectin-1-induced apoptosis. These data strongly suggest that galectin-1-induced apoptosis are clearly distinct from those triggered by Fas engagement.

About the apoptotic signal trigger by cross-linking the T-cell receptor complex, experiments performed whit galectin-1 and T lymphoblastoid cell line that not express CD3, demonstrate that the lectin is capable to activated the death cell program (Pace and Baum, 1997). These results suggest that the mechanism by which galectin-1 can induced apoptosis appears to be distinct from T cell receptor trigger apoptosis.

### Death vs proliferation: Galectin-1 vs Galectin-3

While galectin-1 has been shown to trigger T cell apoptosis (Perillo et al.,1995; Rabinovich et al., 1998; Iglesias et al., 1998a), galectin-3 has been shown to stimulate proliferation (Yang et al., 1996; Iglesias et al., 1998b; Inohara et al., 1998). Similarly to members of the Bcl-2 family, galectins-1 and –3 belong to an additional family of proteins with high sequence homologies but opposite effects on cell survival. The balance between the competing activities of pro-apoptotic proteins such as Bax, Bad and Bak and on the other hand anti-apoptotic proteins such as Bcl-2 and Bcl-xL, determines cell fate (Adams and Cory, 1998). Proteins most similar to Bcl-2 promote cell survival by inhibiting adapters needed for activation of the proteases (caspases) that dismantle the cell, while more distant relatives instead promote apoptosis apparently through mechanisms that include displacing the adapters from the pro-survival proteins. In this sense, the family of Bcl-2 related proteins constitute one of the most relevant apoptotic regulatory gene products acting at the effector stage of apoptosis (Kröemmer, 1997). Hence, it seems meaningful that the interplay between galectins-1 and –3 could also represent an alternative pathway in the normal control of cell homeostasis. To support this hypothesis a striking homology has been found between galectin-3 and Bcl-2 particularly localized in the NWGR domain (Yang, 1996; Akahani et al., 1997).

### Galectin-1 in T cell Adhesion to Extracellular Matrix

Despite the lack of a secretion signal sequence, galectin-1 is secreted into the extracellular millieu, where it recognizes poly-N-acetyl-lactosamine chains on major ECM components, such as laminin (Zho and Cummings, 1993) and fibronectin (Ozeki et al., 1995). By virtue of this recognition, this carbohydrate binding protein has been suggested to act as a modulator of cell-cell and cell-ECM interactions. In collaboration with the laboratory of Dr. Ofer Lider in the Weizmann Institute of Science, Israel, Rabinovich et al. (1999a) has recently shown that galectin-1 (at concentrations below its apoptotic threshold) inhibited the adhesion of human T cells to ECM glycoproteins in a dose and carbohydrate-dependent manner. The inhibition of T-cell adhesion correlated with the ability of this protein to block the re-organization of cell's actin cytoskeleton. Finally, the production of pro-inflammatory cytokines in the context of the ECM was markedly reduced in the presence of this carbohydrate-binding protein. This is the first report as to the role of galectin-1 in T cell adhesion. However, this protein has been shown to promote cell attachment or dettachment on other cell systems such as myoblasts (Cooper et al., 1991), melanocytes (van den Brulle et al., 1995), olfactory neurons (Mahantahappa et al 1994), rhabdomyosarcoma cells (Ozeki et al, 1995) and fibroblasts (Zhou and Cummings, 1993).

### GALECTIN-1 IN T-CELL IMMUNOPATHOLOGY

### Galectin-1, Programmed Cell Death and Autoimmunity: an Attractive Association

Autoimmune disease challenges clinical immunology to set the system right. An autoimmune disease is caused, according to the clonal selection paradigm by aberrant activation of the immune response and loss of central and/or peripheral immune tolerance to self-antigens (Cohen, 1995). The rational answer for harmful activation is to find a way to deactivate the pathogenic lymphocytes. As aforementioned, observations in murine models of systemic autoimmunity and in Canale-Smith syndrome suggest that regulation of lymphocyte apoptosis is crucial to the maintenance of peripheral tolerance (Singer et al., 1994; Fisher et al., 1995). In this context, one should expect that knock out mice for galectin-1 would evidence autoimmune manifestations, such as lupus-like disorders or arthritis, as observed for spontaneous mutations in Fas and FasL in *lpr/lpr* or *gld/gld* mice respectively. However, no important phenotypic changes could be detected in null-mutant mice as regards galectin-1 gene (Poirrier and Robertson, 1993). An exhaustive examination of the immunological system is imperative in these genetically modified mice not only at the central level but also at the periphery to search for potentially harmfull autoaggressive clones and signs of disregulated apoptosis.

Implications of galectin-1 in central and peripheral immune tolerance prompted us to investigate its therapeutic potential in collagen-type II-induced arthritis (CIA) in DBA/1 mice, an experimental model of rheumatoid arthritis (Durie et al., 1994). In collaboration with the laboratory of Dr. Chernajovsky in London, Rabinovich et al. demonstrated by using gene and protein therapy strategies that galectin-1 suppressed arthritis via T cell apoptosis (Rabinovich et al., 1999b). A single injection of syngenic DBA/1 fibroblast engineered to secrete galectin-1 at the day of the disease onset, as well as daily administration of recombinant galectin-1, were both able to abrogate clinical and histopathological manifestations of arthritis. Both treatments resulted in the inhibition of anti-collagen type II (C-II) antibody levels, inhibition of the pro-inflammatory response and a shift towards a Th2-mediated immune response, as judged by the anti-CII IgG isotypes in mice sera at the end of the treatment and the cytokine profile in draining lymph node cells. Finally, clear-cut evidence was provided to show that mice engaged in the gene therapy protocol with galectin-1 increased their susceptibility to antigen-induced apoptosis, providing the first correlation between the apoptotic properties of galectin-1 and its therapeutic potential *in vivo*.

Rheumatoid arthritis (RA) is a common chronic autoimmune disease for which there is not effective therapy capable of preventing long-term progression and joint damage (Feldmann et al., 1996; Chernajovsky et al., 1995). Therefore, effective treatment of arthritis will require the elimination of arthritogenic lymphocytes that initiate and perpetuate joint inflammation, as well as the induction of tissue repair. Hence galectin-1-induced apoptosis could provide for an ideal mechanism using a naturally occurring protein to terminate the autoimmune T-cell attack, preventing the expansion of dominant autoaggressive clones (Vaishnaw et al., 1997). It has been clearly suggested that the extent of apoptosis in RA is inadequate to counteract ongoing proliferation. This imbalance may be explained by the production of cytokines such as IL-1$\beta$, which favor synoviocyte and T-cell proliferation and inhibit susceptibility to apoptosis, possibly associated with increased expression of the Bcl-2 family of proteins (Tsuboi et al., 1996). TNF-$\alpha$ which acts as a potent pro-inflammatory molecule in RA, signals predominantly through the nuclear factor kappa B (NFkB) pathway, promoting the expression of adhesion molecules and recruiting additional cytokines such as GM-CSF and IL-6 in the inflamed joint. Signaling through NF-kB has been suggested to inhibit apoptosis (Fujisawa et al., 1996). Finally, an increase in soluble truncated Fas has been detected in RA synovial fluid thus inhibiting the functional interaction between Fas and FasL (Hasunuma et al., 1997). Altogether, these findings suggest that a disregulated activation of programmed cell death is a critical component of the ethiopathogeny of RA.

Results concerning the role of galectin-1 in suppressing an autoimmune inflammatory process are in agreement with those raised by Levy et al. (1983) in a model experimental autoimmune myasthenia gravis in rabbits and those raised by Offner et al. (1990) in experimental autoimmune encephalomyelitis in Lewis rats.

### CONCLUDING REMARKS

The elucidation of the biochemical pathways and specific proteins that regulate programmed cell death provide a remarkable opportunity to manipulate the life-and death decisions of the cells. The basic understanding and therapeutic manipulation of programmed cell death will have far-reaching implications for the future health of autoimmune disease patients. In this sense, galectins represent an attractive target for biomedical research and clinical intervention. Experimental evidence is now emerging to support the use of galectin-1 not only in the treatment of autoimmune disease, but also in medical strategies aimed at targeting T-cell physiopathology such as the inhibition of transplant rejection, control of graft versus host disease and inhibition of chronic inflammatory processes.

### *Acknowledgements*

We are acknowledged for their contribution to our work to Drs. Clelia Riera, Yuti Chernajovsky, Ofer Lider, Gordon Daily, Hanna Dreja, Cristina Maldonado, Amiram Ariel, Jun Hirabayashi, Carlos Landa, Leonardo Castagna, Mercedes Iglesias, Nidia Modesti and Carlota-Wolfenstein Todel.

This work was supported by grants from "Consejo Nacional de Investigaciones Científicas y Técnicas" (CONICET), "Consejo de Investigaciones Científicas y Tecnológicas de la Provincia de Córdoba" (CONICOR), "Secretaría de Ciencia y Técnica de la UNC" (SeCyT- UNC) and Agencia de Promoción Científica y Tecnológica (FONCIT) PICT N° 02189.

### *References*

Adachi M., Suematsu S., Kondo T., Ogasawara J., Tanaka T., Yoshida N., and Nagata S. (1995). Targeted mutation in the Fas gene cause hyperplasia in the peripheral lymphoid organs and liver. Nature Genetics 11: 294–300.

Adams J.M., and Cory S. (1998). The Bcl-2 protein family: arbiters of cell survival. Science 281: 1322–1326.

Adams D., and Hamilton T.A. (1984). The cell biology of macrophage activation. Annual Review in Immunology 2: 283–293.

Adams L., Kenneth Scott G., and Weinberg C.S. (1996). Biphasic modulation of cell growth by recombinant human galectin-1. Biochimica et Biophysica Acta 1321: 137–144.

Akahani S., Nangia-Makker P., Inohara H, Choi Kim H-R., and Raz A. (1997a) A novel antiapoptotic molecule with a functional BH1 (NWGR) domain of Bcl-2 family. Cancer Research 57: 5272–5276.

Akahani S., Inohara H., Nangia-Makker P., and Raz A. (1997b). Galectin-3 in Tumor metastasis. Trends in Glycoscience and Glycotechnology 9: 69–75.

Aliprantis A.O., Diez-Roux G., Mulder L.C.F., Zychlinsky A., and Lang R.A. (1996). Do macrophages kill through apoptosis? Immunology Today 17: 573–576.

Allen H.J., Sucato D., Gottstine S., Kisailus E., Nava H., Petrelli N., Castillo N and Wilson D. (1991). Localization of endogenous beta-galactoside-binding lectin in human cells and tissues. Tumour Biology 12: 52–60.

Allione A., Wells V., Forni G., Mallucci L., and Novelli F. (1998). β-galactoside-binding protein (β-GBP) alters the cell cycle, up-regulates expression of the α- and β-chains of the IFN-γ receptor, and triggers IFN-γ-mediated apoptosis of activated human T lymphocytes. Journal of Immunology 161: 2114–2119.

Anderson G., Moore N.C., Owen J.J.T., and Jenkison E.J. (1996). Cellular interactions in thymocyte development. Annual Review of Immunology 14: 73–94.

Andersson G., Owen J.J.T., Moor N.C., and Jenkison E.J. (1994). Thymic epithelial cells provide unique signals for positive selection of CD4+CD8+ thymocites in vitro. Journal of Experimental Medicine, 179: 2027–2031.

Barondes S.H., Castronovo V., Cooper D.N.W., Cummings R.D., Drickamer K., Feizi T., Gitt M.A., Hirabayashi J., Hughes C., Kasai K., Leffler H., Liu F., Lotan R., Mercurio A.M., Monsigni M., Pillai S., Poireerr F., Raz A., Rigby P.W.J., Rini J.M., and Wang J.L. (1994a). Galectins: a familly of animal galactoside-binding lectins. Cell 76: 597–598.

Barondes S.H., Cooper D.N.W., Gitt MA and Leffler H. (1994b). Galectins: structure and function of a large family of animal lectins. Journal of Biological Chemistry, 269: 20807–20810.

Baum L.G., Pang M., Perillo N.L., Wu T., Delegeane A., Uittenbogaart C.H., Fukuda M., and Seilhamer J.J. (1995a). Human thymic epithelial cells express an endogenous lectin, galectin-1, which binds to core 2 O-glycans on thymocytes and T lymphoblastoid cells. Journal of Experimental Medicine 181: 877–887.

Baum L.G., Seilhamer J.J., Pang M., Levine W.B, Beynon D. & Berliner J.A. (1995b). Synthesis of an endogenous lectin, galectin-1 by human endothelial cells is up-regulated by endothelial cell activation. Glycoconjugate Journal 12: 63–68.

Blaser C., Kaufmann M., Muller C., Zimmerman C., Wells V., Mallucci L., and Pircher H. (1998). β-galactoside-binding protein secreted by activated T cells inhibits antigen-induced proliferation of T cells. European Journal of Immunology 28: 2311–2319.

Bruner T., Mogil R.J., La Face D.,Yoo N.J., Mahoubl A., Echeverri F., Martin S.J., Force W.R., Lynch D.H., Ware C.F., and Green D.R. (1995). Cell-autonomus Fas (CD95)/ Fas -ligand interaction mediates activation-induced apoptosis in T-cell hybridomas. Nature 373: 441–444.

Chernajovsky Y., Feldmann M., and Main R.V. (1995) Gene theraphy in rheumatoid arthritis via cytokine regulation: future perspectives. British Medical Bulletin 51: 503–516.

Cohen J.J., Duke R.C., Fadok V.A and Sellins K.S. (1992). Apoptosis and programmed cell death in immunity. Annual Review of Immunology, 10:267–293.

Cohen I.R. (1995). Treatment of autoimmune disease: to activate or to deactivate? Chemical Immunology 60: 150–60.

Colnot C., Ripoche M.A., Fowlis D., Cannon V., Scaerou F., Cooper D.N.W. and Poirier F. (1997). The role of galectins in mouse development. Trends in Glycoscience and Glycotechnology 45: 31–40.

Cooper D.N.W., Massa S.M., and Barondes S.H. (1991). Endogenous muscle lectin inhibits myoblast adhesion to laminin. Journal of Cell Biology 115: 1437–1448.

Cooper, D.N. and Barondes S.H. (1990). Evidence for export of a muscle lectin from cytosol to extracellular matrix and for a novel secretory mechanism. Journal of Cell Biology 110: 1681–1691.

Dhein J., Waloczak H., Baumler C., Debatin K.M. and Kramer P.H. (1995). Autocrine T-cell suicide mediated by APO-1 (Fas/CD95). Nature 373: 438–441.

Durie, F.H., Fava R.A., and Noelle R.J. (1994). Collagen-induced arthritis as a model of rheumatoid arthritis. Clinical Immunology and Immunopathology 73: 11–18.

Feldmann M., Brennan F.M., and Main R.V. (1996). Rheumatoid Arthritis. Cell 85, 307–310.

Fisher G.H., Rosenberg F.J., Straus S.E., Dale J.K., Middelton L.A., Yin A.Y., Strober W., Lenardo M.J., and Puck J.M. (1995). Dominant interfering Fas gene mutations impair apoptosis in a human autoimmune lymphoproliferative syndrome. Cell 81: 935–946.

Fujisawa K., Aono H., Hasunuma T., Yamamoto K., Mita S., and Nishioka K. (1996). Activation of transcription factor NF-kB in human synovial cells in response to tumor necrosis factor α. Arthritis and Rheumatism 39: 197–203.

Galle P.R. (1995). Involvement of Apo-1/Fas (CD95) receptor and ligand in liver damage. Journal of Experimental Medicine 182: 1223–1230.

Goldstone S.D., and Lavin M.F. (1991). Isolation of a cDNA clone, encoding a human β-galactoside-binding protein overexpressed during glucocorticoid-induced cell death. Biochemical Biophysical Research Communications 178: 746–750.

Griffith, T.S. (1995). Fas ligand-induced apoptosis as a mechanism of immune privilege. Science 270: 1189–1192.

Hasunuma T., Kayagaki N., Asahara H., Motokawa S., Kobata T., Yagita H., Aono H., Sumida T., Okumura K., and Nishioka K. (1997). Accumulation of soluble Fas in inflamed joints of patients with rheumatoid arthritis. Arthritis and Rheumatism 40: 80–86.

Henkart P.A. (1996). ICE family proteases: mediators of all apoptotic cell death? Immunity 4: 195–201.

Hirabayashi J., and Kasai K. (1988). Complete amino acid sequence of a β-galactoside-binding lectin from human placenta. Journal Biochemistry (Tokyo) 104: 1–4.

Hirabayashi J., and Kasai K. (1993). The family of metazoan metal-independent β-galactoside-binding lectins: structure, function and molecular evolution. Glycobiology 3: 297–304.

Iglesias M.M., Rabinovich G.A., Ambrosio A., Ivanovic V., Sotomayor C.E. and Wolfenstein-Todel C. (1998a). Galectin-1 from ovine placenta: complete primary structure, physicochemical properties and implications in the T cell death. European Journal of Biochemstry 252: 400–407.

Iglesias M.M., Rabinovich G.A., Ambrosio A.L., Castagna L.F., Sotomayor C.E., and Wolfenstein-Todel C. (1998b). Purification of galectin-3 from ovine placenta: developmentally regulated expression and immunological relevance. Glycobiology 8: 59–65.

Inohara H., Akahani S. and Raz A. (1998). Galectin-3 stimulates cell proliferation. E experimental Cell Research 245: 294–302.

Iwatw M., Mukai M., Nakai Y. and Iseki R. (1992). Retinoic acids inhibit activation-induced apoptosis in T cell hybridomas and thymocytes. Journal of Immunology 149: 3302–3308.

Ju S-T, Panka D.J., Cul H., Ettinger R., El-Khatib M., Sherr D.H., Stanger B.Z., and Marsak-Rothstein A. (1995). Fas (CD95)/FasL interactions required for programmed cell death after T-cell activation. Nature 373: 444–448.

Kasai K., and Hirabayashi J. (1996). Galectin: a family of animal lectins that decipher glycocodes. Journal of Biochemistry 119: 1–8.

King L.B., Vacchio M.S., Hunziker R., Margulies D.H., and Ashwell J.D. (1995). A targeted of glucocorticoid receptor antisense transgene increases thymocytes apoptosis and alters thymocytes development. Immunity 5: 647–656.

Kroemer G. (1997). The proto-oncogene Bcl-2 and its rol in regulating apoptosis. Nature Medicine 6: 614–6–20.

Leffler H. (1997). Introduction to galectin. Trends in Glycoscience and Glycotechnology 45: 9–19.

Levi G., Tarrab-Hazdai R., and Teichberg V.I. (1983). Prevention and therapy with electrolectin of experimental autoimmune myasthenia gravis in rabbits. European Journal of Immunology 13: 500–507.

Mahanthappa N.K., Cooper D.N.W., Barondes S.H., and Schwarting G.A. (1994). Rat olfactory neurons can utilize the endogenous lectin L-14, in a novel adhesion mechanism. Development 120: 1373–1384.

Maldonado C., Castagna L.F., Rabinovich G.A., and Landa C. (1999) Immunocytochemical study of the distribution of a 16 kDa galectin in the chicken retina. Investigative Ophthalmol Vis. Sci., in press.

Migliorati G., Nicoletti I., Pagliachi M.C., and Riccardi C. (1992). Glucocorticoid-induced thymocyte apoptosis: inhibition by interleukin-2 and interleukin-4. Pharmacology Research 25: 15–16.

Nagata S. (1997). Apoptosis by death factors. Cell 88: 355–365.

Nishimura Y., Ishi A., Kobayashi Y., Yamasaki Y., and Yonehara S. (1995). Expression and function of mouse Fas antigen on immature and mature T cells. Journal of Immunology 154: 4395–4403.

O'Connell, J., Bennett, M.W., O'Sullivan, G.C., Collins J.K. and Shanahan F. (1999). The Fas counterattack: cancer as a site of immune privilege. Immunology Today 20: 46–52.

Offner H., Celnik B., Bringman T.S., Casentini-Borocz D., Nedwin G.E., and Vandenbark A. (1990). Recombinant human β-galactoside-binding lectin suppresses clinical and histological signs of experimental autoimmune encephalomyelitis. Journal of Neuroimmunology 28: 177–184.

Ogden A.T., Nunes I., Ko K., Wu S., Hines C.S., Wang A.F., Hegde R.S. and Lang R.A. (1998). GRIFIN, a novel lens-specific protein related to the galectin family. Journal of Biological Chemistry 273: 28889–28896.

Osborne B.A. (1996). Apoptosis and the maintenance of homeostasis in the immune system. Current Opinion of Immunology 8: 245–354.

Ozeki Y., Matsui T., Yamamoto Y., Funahashi M., Hamako J., and Titani K. (1995). Tissue fibronectin is an endogenous ligand for galectin-1. Glycobiology 5: 255–261.

Pace K.E., and Baum L.G. (1997). Induction of T lymphocyte apoptosis: a novel functions for galectin-1. Trends in Glycoscience and Glycotechnology 45: 21–29.

Perillo N.L., Pace K.E., Seilhamer J.J., and Baum L.G. (1995). Apoptosis of T cells mediated by galectin-1. Nature 378: 736–739.

Perillo N.L., Oittenbogaart C.H., Nguyen J.T. and Baum L.G. (1997). Galectin-1 and endogenous lectin produced by human thymic epithelial cells, induces apoptosis of the human thymocytes. Journal of Experimental Medicine 97: 1851–1859.

Poirrier F., and Robertson E.J. (1993). Normal development of mice carrying a null mutation in the gene encoding the L14 S-type lectin. Development 119: 1229–1236.

Rabinovich G.A., Castagna L.F., Landa C.A., Riera C.M., and Sotomayor C.E. (1996). Regulated expression of a 16-kd galectin-like protein in activated rat macrophages. Journal of Leukocyte Biology 59: 363–370.

Rabinovich G.A., Iglesias M.M., Wolfenstein-Todel C., Castagna L.F., Modesti N, Riera C.M., and Sotomayor C.E. (1998). Rat activated macrophages produce a galectin –1-like protein which induces apoptosis of T cells: Biochemical and functional characterizations. Journal of Immunology. 160:4831–4840.

Rabinovich G.A., Modesti N., Castagna L.F., Landa C., Riera C.M., and Sotomayor C.E. (1997). Specific inhibition of lymphocyte proliferation and induction of apoptosis by CLL-I, a β-galactoside-binding lectin. Journal of Biochemistry 122: 365–373.

Rabinovich, G.A. (1999). Galectins: an evolutionarily conserved family of animals proteins with multifunctional properties: a trip from the gene to the clinical therapy. Cell Death and Differentiation, 6: 711-722.

Rabinovich, G.A., Ariel, A., Hershkoviz. R., Hirabayashi, J., Kasai, K.I., and Lider, O. (1999a). Specific inhibition of T-cell adhesion to extracellular matrix and pro inflammatory cytokine secretion by human recombinant galectin-1. Immunology, 97: 100-106.

Rabinovich G.A., Daily G., Dreja H., Taylor H., Hirabayashi J., Riera C.M., and Chernajovsky Y. (1999b). Protein and gene delivery of galectin-1 suppress arthritis via T cell apoptosis. Journal of Experimental Medicine, 190: 385-398.

Rabinovich G.A., Aoki M.P., Maldonado C., Yranzo N.L and Sotomayor C.E. (1999c). Regulated secretion and ultrastructural distributions of RMGal, a pro-apoptotic galectin-1-like protein. Submitted.

Raff M.C. (1992). Social controls on cell survival and cell death. Nature 356:397–400.

Rieux-Laucat F., Le Deist F., Hivroz C., Roberts I.A.G., Debatin., Fischer A., and De Villartay J.P. (1995). Mutation in Fas associated with human lymphoproliferative syndrome and autoimmunity. Science 268: 1347–1349.

Russell J.H., and Wang R. (1993). Autoimmune gld mutation uncouples suicide and cytokine/ proliferation pathway in activated mature T cells. European Journal of Immunology 23: 2379–2382.

Russell J.H., Rush B.,Weaver C., and Wang R. (1993). Mature T cell of autoimmune lpr/lpr mice have a defect in antigen-stimulated suicide. Proceedings of Natural Academy of Sciences USA 90: 4409–4413.

Sandford G.L., and Harris-Hooker S. (1990). Stimulation of vascular cell proliferation β-galactoside-binding lectins. FasEB Journal 4: 2912–2918.

Schwartzman R.A., and Cidlowski J.A. (1993). Apoptosis: the biochemistry and molecular biology of programmed cell death. Endocrinology Review 14: 133–151.

Singer G.G., Carrera A.C., Marshak-Rothstein A., Martinez A.C., and Abbas A.K. (1994). Apoptosis, Fas and systemic autoimmunity: the MRL lpr/lpr model. Current Opinion of Immunology 6: 913–920.

Singer G.G. and Abbas A.K. (1994). The Fas antigen is involved in pheripheral but not thymic delection of T lymphocytes in T cell receptor transgenic mice. Immunity 1:365–371.

Sprent J., Lo D., Gao E.K., and Ron Y. (1988).T cell selection in the thymus. Immunology Review 101: 172–190.

Strand S., and Galle R.P. (1998). Immune evasion by tumors: involvement of CD 95 (APO-1/Fas) system and its clinical implications. Molecular Medical Today 2: 63–68.

Streilin, J.W. (1993). Tissue barriers, immunosuppressive microenvironments, and privileged sites: the eyes's point of view. Regional Immunology 5: 253–268.

Su Z.Z., Lin J., Shen R., Fisher P.E., Goldestein N.I., and Fisher P.B. (1996). Proceedings Natural Academy of Sciences USA 93: 7252–7257.

Suda T., Okazaki T., Naito Y., Yokoto T., Arai N., Ozaki S., Nakao K., and Nagata S. (1995). Expression of the Fas ligand in cells of the T cell lineage. Journal of Immunology 154: 3806–3813.

Surh C.D., and Sprent J. (1994). T-cell apoptosis detected in situ during positive and negative selection in the thymus. Nature 372: 100–103.

Takayama H., and Sitkovsky M.V. (1989). Potential use of an antagonist of cAMP-dependent protein kinase to block inhibition and modulate T-cell receptor trigger activation of cytotoxic T-lymphocytes. Journal of Pharmaceutical Science; 78: 8–10.

Tanaka M., Suda T., Takanahashi T., and Nagata S. (1995). Expression of the functional soluble forms of the human Fas ligand in activated lymphocytes. European Molecular Biology Organization Journal 14: 1129–1135.

Thompson C.B. (1995). Apoptosis in the pathogenesis and treatment of disease. Science 267: 1456–1462.

Tsuboi M., Eguchi K., Kawakami A., Matsuoka N., Kawabe Y., Aoyagi T., Maeda K., and Nagataki S. (1996) Fas antigen expression on synovial cells was downregulated by interleukin-1 beta. Biochemistry and Biophysic Resesarch Communications 218:280–285.

Vacchio M.S., Papadopulous V., and Aswell J.D. (1994). Steroid production in the thymus: implications for thymocites selection. Journal of Experimental Medicine 179: 1835–1846.

Vaishnaw A.K. Mc Nally J.D., and Elkon K.B. (1997) Apoptosis in the rheumatic diseases. Arthritis and Rheumatism 40: 1917–1927.

van den Brüle F.A., Buicu C., Baldet M., Sobel M.E., Cooper D.N.W, Marschal P., and Castronovo V. (1995). Galectin-1 modulates human melanoma cell adhesion to laminin. Biochemical Biophysical Research Communications 209: 760–767.

van Parijs L., and Abbas A.K. (1998). Homeostasis and self-tolerance in the immune system: turning lymphocytes off. Science 280: 243–248.

van Parijs L., Peterson D.A., and Abbas A.K. (1998). The Fas/ Fas ligand pathway and Bcl-2 regulate T cell responses to model self and foreign antigens. Immunity 8: 265–274.

Vaux D.L., Haecker G. and Strasser A. (1994). An evolutionary perspective on apoptosis. Cell 76: 777–779.

Wang J., Zheng L., Lobito A., Ka-Ming Chan F., Dale J.K., Sneller M., Jaffe E., Puck J.M., Straus S.E., and Lenardo J. (1999). Inhereted human caspase-10 mutation underlies defective lymphocyte apoptosis and dendritic cell apoptosis in autoimmune lymphoproliferative syndrome, type II. Cell, 1999, in press.

Wells V., and Mallucci L. (1991). Identification of autocrine negative growth factor: mouse β-galactoside-binding protein is a cytostatic factor and cell growth regulator. Cell 64: 91–97.

Wyllie A.H. (1980). Glucocorticoid induced thymocyte apoptosis is associated with endogenous endonucluease activation. Nature, 284: 555–556.

Yang R.Y., Hsu D.K., and Liu F.T. (1996). Expression of galectin-3 modulates T cell growth and apoptosis. Proceedings Natural Academy of Sciences USA, 93: 6737–6742.

Zhou Q., and Cummings R.D. (1993). L-14 lectin recognition of laminin and its promotion of in vitro cell adhesion. Archives of Biochemistry and Biophysics 300: 6–17.

